# Effects of dietary dimethylglycine supplementation on laying performance, egg quality, and tissue index of hens during late laying period

**DOI:** 10.1016/j.psj.2021.101610

**Published:** 2021-11-20

**Authors:** Hong Yao, Yan Hu, Qiang Wang, Yijian Zhang, Kaiqing Rao, Shourong Shi

**Affiliations:** ⁎ Poultry Institute, Chinese Academy of Agriculture Science, Yangzhou, Jiangsu, 225125, China; † Jiangsu Co-Innovation Center for the Prevention and Control of Important Animal Infectious Disease and Zoonose, Yangzhou University, Yangzhou, 225009, China; ‡ College of Animal Husbandry and Veterinary, Southwest Minzu University, Chengdu, 610041, China

**Keywords:** dimethylglycine, laying performance, egg quality, late laying period

## Abstract

In this study, the effects of 5 graded dietary levels (0.025, 0.05, 0.075, 0.1, and 0.125%) of dimethylglycine (**DMG**) were studied in laying hens during the late laying period (71–78 wk). Graded doses of DMG from 0.025 to 0.125% in the diet produced quadratic positive (*P* < 0.05) responses in the laying rate, egg-feed ratio, yolk color, grade follicular weight, and the number of large white follicles and linear positive (*P* < 0.05) responses in average egg weight, and the number of large white follicles. With 0.1% DMG, the laying rate and egg-feed ratio improved (*P* < 0.05), and the abdominal fat percentage decreased. Considering the laying performance under the conditions used in this study, the best-fit model for the DMG requirements of laying hens was estimated to range from 0.049 to 0.065% DMG during the late laying period based on a regression analysis. The addition of DMG did not affect the total cholesterol (**TCH**) and triglyceride (**TG**) contents in the plasma of laying hens; however, it significantly reduced the abdominal fat rate. DMG may change the course of lipid deposition in laying hens during the late laying period. In conclusion, supplementation with DMG can improve the laying rate and follicles development of laying hens.

## INTRODUCTION

Eggs are a valuable source of dietary proteins, lipids, amino acids, and minerals (Liu et al., 2018). Longer laying periods for commercial laying hens make better use of chicken house equipment and reduce the cost of brooding. The age of commercial laying hens has been extended beyond 72 wk to 80 wk. After the peak laying period and the corresponding decrease in yolk synthesis and accumulation, the number of follicles recruited into the preovulatory hierarchy decreases (Lillpers and Wilhelmson, 1993), resulting in a decrease in the laying performance of hens. The decrease in laying performance is mainly manifested in a decrease in the laying rate; a decline in egg quality, such as eggshell quality or Haugh unit, and an increase in the egg-feed ratio (Zakaria et al., 1983; Joyner et al., 1987). Moreover, lipid metabolism disorders, and visceral fat and abdominal fat deposition increase with age, which further affects the health and laying performance of hens (Shini et al., 2019; Xie et al., 2019). However, it is fundamentally difficult to use genetics and other fields of biotechnology to improve the production performance of laying hens. Genetic selection for increasing egg production persistency currently allows hens to keep for a longer production period. It is expected that, in the next few years, commercial layers will produce approximately 500 eggs in a production cycle of 100 wk without molting, albeit with potential adverse effects on egg quality (Bain, Nys et al. 2016).

In recent years, many studies in mammals have found that supplementing methyl donors such as choline, betaine, and methionine can effectively prevent and alleviate fatty liver syndrome by regulating fat metabolism (Albuquerque et al., 2017; Mou et al., 2018). Betaine can directly participate in fat metabolism, amino acid transfer and regeneration, purine and pyrimidine biosynthesis, and other biochemical processes (Joselit et al., 2018; Abu Ahmad et al., 2019; Zhang et al., 2019). Methionine content can affect the laying rate, egg weight, and lipid deposition (Burley and Patterson 2017; Li et al., 2018; Li et al., 2019). Betaine can partly replace methionine and play the role of methyl supply, so as to play the role of saving methionine (Abobaker et al., 2017). Supplementation with betaine reduced the cost of feed, and the consumption of methionine methyl donors; it also increased the protein synthesis efficiency of methionine, and improved the laying performance (Hao et al., 2017). Dimethylglycine (**DMG**) is a trans-methyl product of betaine formed under the action of betaine-homocysteine methyltransferase (**BHMT**), which takes place in the choline to glycine betaine pathway (Tonda and Hart, 1992; Cools et al., 2010). DMG is a natural substance in animal and plant metabolic pathways, and it occurs in food, such as cereals, beans, and liver. Betaine and DMG can be used for the remethylation of homocysteine to methionine (Kharbanda, 2013). DMG can also provide methyl to methionine to produce homocysteine, forming a closed cycle in which DMG acts as a methyl donor, thereby improving the utilization rate of methyl. However, no pieces of data are available regarding the effect of DMG on the laying performance of hens.

Therefore, this study aimed to investigate the effects of dietary supplementation of DMG, at different levels, on the laying performance of hens during the late laying period. We also sought to explore whether exposure to DMG affects egg quality and follicle classification.

## MATERIALS AND METHODS

### Birds and Experimental Design

Four hundred and thirty-two Hy-line laying hens were raised at the Poultry Institute of the Chinese Academy Agricultural Sciences (Yangzhou, 32^°^31′17.56 North, 119^°^30′30.97 East, 6.4 m above sea level), from November 2019 to January 2020 (winter, the outdoor temperature was 0–15°C). Birds were allocated randomly into control and DMG groups (6 replicates/group, 12 hens/replicate) at 70 wk of age. The average laying rate before the official trial was 92%. Every hen was confined individually with a cage size of 24 × 40 × 40 cm and each of the 12 birds (12 cages) shared a common feed trough between them, forming one experimental unit. Cages were located in a ventilated room with a temperature range of 18°C to 20°C and 15.5 h/d of illumination with an incandescent lamp of 15 lx/m^2^. Hens were provided ad libitum access to water with nipple drinkers and feed troughs. They were also given a basal (Control) or DMG-supplemented diet for 8 wk. The study was conducted according to the Regulations of the Experimental Animal Administration issued by the State Committee of Science and Technology of the People's Republic of China. The animal use protocol was approved by the Animal Care and Use Committee of the Poultry Institute, Chinese Academy of Agriculture Science (No. CNP20191108).

### Experimental Diet and Groups

The basal corn-soybean meal diet used in the experiment (Table 1) was based on the Hy-Line Brown Commercial Management Guide 2018; one group was fed a basal diet only (control, CON), and the treatment groups were fed the basal diet that containing DMG. The treatment groups consisted of a basal diet supplemented with 0.025 (abbreviated as N1), 0.05 (abbreviated as N2), 0.075 (abbreviated as N3), 0.10 (abbreviated as N4), and 0.125% (abbreviated as N4) DMG (DMG supplied by Zhendong Chemical Co. Ltd., Kunshan, China; purity 97%).

**Table 1:** Ingredients and nutrient levels of the diet.^1^

Dietary ingredients	Content (%)
Corn	62.03
Soybean meal (43% HP)	27.0
Soybean oil	0.75
Limestone	8.5
Dicalcium phosphate	1.0
DL-methionine	0.1
NaCl	0.3
70% Choline chloride	0.09
Mineral premix^2^	0.2
Vitamin premix^3^	0.03
Total	100
Nutrients levels 4	
Metabolizable energy (Mcal/kg)	2.711
Crude protein	16.547
Calcium	3.444
TP	0.563
Lysine	0.885
Methionine	0.381
Cystine	0.280

1 Values are expressed on an air-dry basis.

2 Mineral premix provided the following per kilogram of the diet: Cu, 9.97 mg; Fe, 59.83 mg; Zn, 47.86 mg; Mn, 59.83 mg; Se, 0.30 mg, I, 0.40 mg; Co, 0.30 mg.

3 Vitamin premix provided the following per kilogram of the diet: vitamin A, 11,396 IU; vitamin D3, 3,133 IU; vitamin E, 17.09 IU; vitamin K3, 1.71 mg; vitamin B1, 1.99 mg; vitamin B2, 6.27 mg; vitamin B6, 3.13 mg; vitamin B12, 0.02 mg; folic acid, 1.42 mg; biotin, 0.03 mg; niacin, 28.49 mg; calcium pantothenate, 9.97 mg.

4 Metabolizable energy is a theoretical value; calcium, phosphorus and amino acid content are measured values.

### Sample Collection and Analytical Determination

#### Laying Performance

Laying performance parameters, included the egg number and the weight, egg production, and average daily feed intake (**ADFI**). Average egg weight was calculated as the mean weight of all eggs from each replicate, and the egg-feed ratio was recorded every week. The AFDI was determined weekly by subtracting the final feed weight of each replicate from the initial feed weight.

#### Egg Quality

Eggs laid on the last day of the dietary treatment were collected. At the end of the experiment, 144 normal eggs were randomly selected (n = 24 eggs/group, 4 eggs/ replicate) from each dietary treatment to evaluate egg quality traits, including egg weight, egg yolk weight, shell weight, eggshell strength, and shell thickness. Eggshell strength, yolk color, and the Haugh unit were determined using an EA-01 egg analyser (ORKA Food Technology, Ltd., Ramat Hasharon, Israel). Shell thickness was determined using a shell thickness gauge (B26976, 0–20 mm, digital display external micrometer, Guanglu Measuring Instrument Co., Ltd., Guilin, P. R. China) at 3 different locations (bottom, middle, and top of the egg).

### Plasma Lipid Content

At the end of the dietary treatment, the wing vein blood samples (approximately 2 mL) from 36 birds (1 bird from each replicate, 6 birds per group) were placed into tubes containing EDTA-Na2 (an anticoagulant). The samples were centrifuged at 3,000 rpm/min for 10 min (Cence DL-5M Low-speed freezing centrifuge, Hunan Xiang Yi Laboratory Instrument Development Co., Ltd) to collect plasma for blood lipid content determination, which included total cholesterol (**TCH**) and triglyceride (**TG**) contents. The plasma parameters were measured with a microplate reader (infinite M200 pro, TECAN, Männedorf, Switzerland) using commercial kits (Triglyceride GPO-PAP Assay kit, A110-1-1 and Technologies COD-PAP Assay kit, A111-1, Nanjing Jiancheng Institute of Bioengineering, Nanjing, Jiangsu, P. R. China) following the manufacturer's instructions.

### Tissue Index

All birds, from which blood samples were collected, were weighted and slaughtered by neck bleeding. The skin was cut along the epidermis near the cloaca, and the epidermis was torn to the neck to completely expose the chest and abdomen. The abdominal fat, spleen and liver were weighed, and the follicles were graded and counted. Follicles are usually divided into 3 types according to their size and color: >10 mm, grade follicles; 4 to 10 mm, small yellow follicles; 2 to 4 mm, large white follicles (Lovell et al., 2003).

### Statistical Analysis

Statistical analyses were conducted using SPSS (version 26.0; SPSS Inv., Chicago, IL). Differences in DMG diet supplementation were tested using one-way analysis of variance (**ANOV**A) for independent samples. Univariate analysis of the general linear model was used to test the main effects of the 5 DMG doses. Regressions of estimation curves were used to compare the best-fit quadratic and linear models for these objective traits. All data are presented as the means with pooled SEM values. Statistical significance was set at *P* < 0.05.

## RESULTS

### Laying Performance

At 71 to 74 wk, dietary DMG administration had no significant effects (*P* > 0.05) on laying performance parameters, such as egg-laying rate, egg mass, ADFI, average egg weight, and egg-feed ratio (Table 2).

**Table 2:** Effects of dietary dimethylglycine (DMG) supplementation on laying performance of late laying period hens.^1^

Items	Groups^2^						SEM	Probability		
	CON	N1	N2	N3	N4	N5		DMG	Linear	Quadratic
71–74W										
Laying rate (%)	93.0^ab^	93.1^ab^	91.2^a^	91.2^ab^	93.5^b^	93.0^ab^	0.3	0.159	0.822	0.069
Egg mass (g/day/hen)	55.7	56.4	55.3	55.6	56.9	56.7	0.3	0.590	0.342	0.460
ADFI (g/d)	109.9	110.9	110.4	111.0	109.3	110.2	0.4	0.783	0.714	0.563
Average egg weight (g)	60.7^ab^	60.6^ab^	60.8^a^	60.8^a^	61.9^b^	61.0^ab^	0.2	0.390	0.162	0.925
Egg-feed ratio^3^	1.958^ab^	1.942^ab^	1.997^b^	1.982^ab^	1.920^a^	1.943^a^	0.009	0.162	0.127	0.135
75–78W										
Laying rate (%)	91.28^ab^	90.95^ab^	89.01^a^	92.48^b^	92.50^b^	92.68^b^	0.28	0.060	0.186	0.138
Egg mass (g/day/hen)	56.4 ^b^	55.0^ab^	54.0^a^	56.9^bc^	57.2 ^c^	57.2^bc^	0.3	0.001	0.062	0.002
ADFI (g/d)	113.04	113.90	113.21	112.02	111.60	113.33	0.28	0.762	0.636	0.769
Average egg weight (g)	61.55^ab^	60.53^a^	61.37^ab^	60.97^ab^	61.93^b^	61.74^ab^	0.11	0.018	0.021	0.088
Egg-feed ratio^3^	2.022^ab^	2.094^c^	2.078^bc^	1.988^a^	1.952^a^	1.980^a^	0.009	<0.001	<0.001	0.060
71–78W										
Laying rate (%)	91.53^ab^	92.10^ab^	90.21^a^	91.78^ab^	93.17^b^	92.86^b^	0.20	0.007	0.069	0.042
Egg mass (g/day/hen)	55.82^ab^	56.32^ab^	54.93^a^	55.99^ab^	56.88^b^	56.94^b^	0.24	0.123	0.143	0.110
ADFI (g/d)	110.84	111.46	111.64	111.01	110.90	111.63	0.23	0.437	0.373	0.426
Average egg weight (g)	60.99^ab^	60.56^a^	61.07^a^	61.00^ab^	61.92^b^	61.32^ab^	0.08	0.006	0.003	0.297
Egg-feed ratio^3^	1.983^bc^	2.002^bc^	2.032^c^	1.985^bc^	1.926^a^	1.960^ab^	0.007	0.002	0.009	0.043

Note: In the same line, the values with the same letter are not significantly different for all possible combinations of these different groups (*P* > 0.05), and values with different letters are significantly different for all possible combinations of these different groups (*P* <0.05).

1 results are means with n = 6 per treatment, the same as tables below.

2 N1 means supplement 0.025% DMG to the basal diet; N2 means supplement 0.05% DMG to the basal diet; N3 means supplement 0.075% DMG to the basal diet; N4 means supplement 0.1% DMG to the basal diet; N5 means supplement 0.125% DMG to the basal diet.

3 Egg-feed ratio (percent of the average daily feed intake relative to the egg-weight gain).

At 75 to 78 wk, egg mass, average egg weight, and egg-feed ratio were significantly affected by DMG addition (Table 2, shown as total *P*-DMG = 0.001, 0.018, and < 0.001, respectively). Egg mass significantly fit a quadratic model (*P* = 0.002, Y = 58.533+1.377X-0.129X^2^, estimated optimal dose required was 0.053% DMG). Dietary DMG had significant effects on average egg weight (*P* = 0.021, linear model: Y = 60.89+5.86X) and egg-feed ratio (*P* < 0.001, linear model: Y = 2.07-0.71X).

At 71 to 78 wk, dietary DMG had significant effects on average laying rate, average egg weight, and egg-feed ratio (Table 2, shown as total *P*-DMG = 0.007, 0.006, and 0.002, respectively). The laying rate significantly fit a quadratic model (*P* = 0.042, Y = 92.08+221.95X-19.34X^2^), indicating that 0.057% DMG was the optimal level of supplementation. DMG dietary treatment had significant linear effects on average egg weight (*P* = 0.003, linear model: Y = 60.74+5.44X) and egg-feed ratio (*P* =0.009, linear model: Y = 2.01-0.41X). Moreover, DMG dietary treatment had significant quadratic effects on the egg-feed ratio (*P* = 0.043, quadratic model: Y = 1.99-8.88X+0.70X^2^, estimated optimal dose required was 0.063% DMG). Except for the laying rate (*P* = 0.069), age and DMG had a synergistic effect on average egg weight (*P* = 0.009) and egg-feed ratio (*P* < 0.001).

### Egg Quality

As shown in Table 3, yolk color at 78 wk increased quadratically (*P* = 0.030) and significantly fit a quadratic model (Y = 5.08+9.30X-95.17X^2^), indicating that 0.049% DMG was the optimal level of supplementation (Table 3). The N2 dietary treatment increased shell weight and shell thickness (compared to the CON group, *P* = 0.035 and 0.016, respectively). Compared to the CON group, the N2, N3, and N5 dietary treatments increased the yolk weight (*P* = < 0.001, 0.032, and 0.004, respectively).

**Table 3:** Effects of dietary dimethylglycine (DMG) supplementation on egg quality of late laying period hens.

Items	Groups						SEM	Probability		
	CON	N1	N2	N3	N4	N5		DMG	Linear	Quadratic
Egg weight (g)	61.43	59.51	62.07	62.14	60.38	60.84	0.32	0.109	0.964	0.417
Shell weight (g)	5.996	6.050	6.258	6.104	5.896	6.121	0.037	0.117	0.991	0.312
Shell percentage^1^ (%)	9.772	10.060	10.226	9.838	9.721	10.070	0.063	0.130	0.942	0.531
Shell strength (pa)	3.875	4.019	4.127	4.052	3.981	4.086	0.054	0.833	0.821	0.802
Shell thickness (mm)	0.348	0.359	0.370	0.359	0.350	0.360	0.002	0.065	0.595	0.104
Shape index^2^ (%)	76.21	77.74	76.86	76.28	76.44	76.44	0.26	0.562	0.560	0.583
Yolk color	4.85	5.49	5.61	4.88	4.90	4.92	0.08	0.004	0.139	0.030
Yolk weight (g)	16.18	16.39	17.00	16.81	16.30	16.86	0.11	0.157	0.180	0.252
Yolk percentage^3^(%)	27.05	27.55	27.47	26.99	26.85	27.50	0.17	0.764	0.939	0.906
Albumen weight (g)	38.50	37.19	39.00	39.43	38.50	38.04	0.28	0.276	0.191	0.171
Albumen percentage^4^(%)	63.20	62.26	62.86	63.36	63.42	62.43	0.20	0.420	0.975	0.602
Albumen height	6.26	5.58	5.79	6.07	6.01	6.08	0.09	0.281	0.197	0.234
Haugh unit	77.87	72.72	73.39	75.96	75.53	76.12	0.71	0.317	0.217	0.309

1 Shell percentage: shell weight/egg weight.

2 Shape index: egg transverse span/egg longitudinal span.

3 Yolk percentage: yolk weight/egg weight.

4 Albumen percentage: albumen weight/egg weight.

### Plasma Lipid Content

At 78 wk, dietary DMG administration had no significant effects on TG and TCH content in the plasma (*P* > 0.05) (Table 4, shown as total *P*-DMG). Compared to N2, the TG levels in the plasma of N3 and N4 showed a decreasing trend (*P* = 0.079, 0.034), and the N5 was significantly decreased (*P* = 0.021).

**Table 4:** Effects of dietary dimethylglycine (DMG) supplementation on plasma lipid content of late laying period hens.

Items	Groups						SEM	Probability		
	NC	N1	N2	N3	N4	N5		DMG	Linear	Quadratic
TG (mmol/L)	12.0	12.8	14.2	11.8	12.1	11.1	0.4	0.646	0.412	0.279
TCH (mmol/L)	4.1	3.9	4.3	3.8	3.5	3.9	0.1	0.570	0.384	0.891

### Tissue Index

As shown in Table 5, grade follicular weight at 78 wk significantly fit a quadratic model (Y = 37.03-214.82X+1961.09X^2^), indicating that 0.055% DMG was the optimal level of supplementation. Dietary DMG had significant effects on the number of large white follicles (linear *P* = 0.037, linear model: Y = 45.19-3.05X; quadratic *P* = 0.021, quadratic model: Y= 46.68-92.33X+714.29X^2^, estimated optimal dose required was 0.065%). Compared to the CON group, the N4 and N5 dietary treatments decreased the abdominal fat percentage (*P* = 0.001 and < 0.001, respectively).

**Table 5:** Effects of dietary dimethylglycine (DMG) supplementation on the tissue index of late laying period hens.

Items	Groups						SEM	Probability		
	CON	N1	N2	N3	N4	N5		DMG	Linear	Quadratic
Body weight (g)	1884.0	1938.7	1922.5	2005.8	2053.3	1912.3	29.8	0.659	0.183	0.686
Abdominal fat weight (g)	76.8	71.0	63.6	52.1	72.1	56.3	4.2	0.621	0.269	0.483
Abdominal fat percentage (%)	4.09	3.71	3.25	2.76	3.41	2.71	0.20	0.367	0.059	0.546
Spleen weight (g)	2.22	2.10	2.15	2.25	2.32	2.38	1.26	0.150	0.095	0.726
Spleen index (%)	0.113	0.109	0.113	0.113	0.113	0.126	0.004	0.868	0.920	0.959
Liver weight (g)	43.7	39.2	35.1	39.6	46.4	38.4	0.1	0.539	0.606	0.631
Liver index (%)	2.068	2.034	1.840	2.066	2.059	2.011	0.039	0.523	0.984	0.455
Grade follicular weight (g)	38.6	32.2	32.2	36.4	39.9	33.2	1.3	0.115	0.321	0.018
Grade follicles	5.33	5.00	5.00	5.00	5.17	4.83	0.09	0.644	0.267	0.739
Grade follicles weight/number (g)	7.22	6.27	6.67	7.05	7.59	6.84	0.17	0.325	0.257	0.166
Small yellow follicles	12.50	18.67	11.67	12.33	14.17	13.00	0.79	0.109	0.075	0.039
Large white follicles	47.67	47.17	32.83	52.83	45.17	44.33	1.94	0.066	0.037	0.021

## DISCUSSION

This study found that dietary DMG supplementation significantly increased laying performance, similarly to previous results, showing recorded that dietary DMG supplementation could enhance the growth performance of broilers (Kou et al., 2015), and improve the final body weights and production value of broilers (Chalvatzi et al., 2020). In addition, we found that there was no difference in the effects of 0.1 and 0.125% DMG dietary supplementation from 71 to 78 wk on laying rate, which was consistent with the results of previous studies on broilers (Yang et al., 2015) and laying hens (Abobaker et al., 2017). The effects of DMG or betaine on the growth or laying performance of birds were quadratic and not dose-dependent. Betaine and homocysteine synthesize methionine, reduce methionine consumption as a methyl donor, and increase the contents of internal essential amino acids in animals (Li et al., 2014). Comparative experiments of betaine and methionine with laying hens have demonstrated that betaine and methionine supplementation have similar effects on the laying rate (Dänicke et al., 2006; Hao et al., 2017). Previous experiments have shown that the effect of methionine supplementation on laying hens has some of the same effects as DMG and betaine supplementation (Song et al., 2014; Liu and Wang, 2017; Geng et al., 2018). This finding confirms that the effects of exogenous methyl donors on laying performance were quadratic and not dose-dependent.

DMG dietary treatment had a significant effect on the laying rate, average egg weight, and the egg-feed ratio of laying hens from 71 to 78 wk. Among the doses of DMG dietary treatments examined in this study, 0.057% DMG optimized the laying rate. Although the addition of DMG to the diet had no effect on the egg mass in wk 71 to 78, it was found that the supplement had a quadratic linear effect on the egg production in wk 75 to 78, wherein the optimal addition was 0.053%. This may be related to the length of the treatment time. Compared to 0.1% DMG, the 0.05% DMG diet decreased the laying rate. However, 0.05% DMG supplementation improved egg quality at 78 wk. Previous studies have confirmed that half an hour after the previous egg is laid, the ovary begins to discharge the next egg and the formation time of the eggshell accounts for 4/5 of the entire egg formation time (Xiang, 1983; Dai, 2019). If the egg stays in the uterus for a long time, and the eggshell deposits better, thereby enhancing eggshell quality (Zhang, 2015). Although the laying rate observed under 0.05% DMG was lower than that under 0.1% DMG, the shell weight, shell thickness, yolk color, and yolk weight were significantly improved by 0.05% DMG. Previous results indicated that the yolk color of laying hens gradually decreased at the later stage of laying (Zhang et al., 2010). In this experiment, 0.05% DMG improved the yolk color of laying hens during the late stage of laying and betaine had no effect on yolk color (Chen et al., 2018). However, whether or not DMG is involved in pigment deposition in eggs requires further study. The data indicated that egg quality was optimized under 0.049% DMG. Considering the laying performance and feeding cost, the best-fitting models of the DMG requirements of laying hens were those with relatively lower levels of DMG supplementation during the late laying period.

Methyl donors can regulate lipid metabolism and affect metabolic outcomes (Mittelstraß and Waldenberger 2018; Zhong et al., 2018). Betaine inhibits hepatic fat accumulation, and increases mitochondrial content and activity, which suggests that it is involved in the regulating lipid metabolism (Zhang et al., 2019). Furthermore, in ovo betaine injection alleviates CORT-induced hepatic cholesterol deposition (Hu et al., 2017). The fat metabolism function of laying hens is closely related to egg production because in the process of egg formation, yolk production requires a large amount of lipid deposition (Burley et al., 1993). In this study, the TG content in the plasma was higher with 0.05% DMG than with 0.1% DMG and 0.125% DMG. The laying rate with 0.05% DMG was significantly lower than with 0.1% DMG and 0.125% DMG. High-dose DMG may improve the laying rate of laying hens during the late laying period by affecting lipid turnover. Previous studies in poultry have found that betaine supplementation may positively affect lipid metabolism by increasing fatty acid catabolism, thereby playing a key role in reducing carcass fat deposition (García-Lezana et al., 2018). Incremental levels of betaine decreased LDL, TG, and TCH in broiler serum and effectively reduced abdominal fat deposition in a dose-dependent manner (Hu et al., 2017). This may be why high doses of DMG resulted in lower abdominal fat in laying hens during the late laying period.

There is abundant evidence that the supplementation of methyl donors in mammals has positive physiological effects on health (Sun et al., 2016; Davis et al., 2017; Riis Poulsen et al., 2018), growth (Jin et al., 2018; Lan and Kim, 2018; Ma et al., 2020), and reproduction (Lugar et al., 2018; Robinson et al., 2018). It has also been found to affect the endocrine status and improve lipid metabolism (Zeisel, 2013; Wang et al., 2015; Kang et al., 2018). Similarly, our results showed that oral DMG supplementation in laying hens enhanced grade follicular weight and the number of large white follicles; we also found that the optimal performance of these 2 indexes required 0.055% DMG and 0.065% DMG, respectively. Supplementation with 0.1% DMG and 0.125% DMG decreased abdominal fat weight and abdominal fat percentages. Supplementation with DMG had beneficial effects on the laying performance and the deposition of fat in laying hens, which requires a methyl donor.

In summary, this study provides the evidence that dietary DMG has a positive effect on laying rate, average egg weight, egg-feed ratio, and yolk color of hens during the late laying period. Moreover, the best-fit model for the DMG requirements of the late laying period hens, regarding the optimal range of the laying rate, egg-feed ratio, follicles, and yolk color, were estimated from regression analysis to be between 0.049 and 0.065%.
